# Role of hippocampal location and radiation dose in glioblastoma patients with hippocampal atrophy

**DOI:** 10.1186/s13014-021-01835-0

**Published:** 2021-06-22

**Authors:** Clara Le Fèvre, Xue Cheng, Marie-Pierre Loit, Audrey Keller, Hélène Cebula, Delphine Antoni, Alicia Thiery, Jean-Marc Constans, François Proust, Georges Noel

**Affiliations:** 1 Department of Radiation Oncology, UNICANCER, Paul Strauss Comprehensive Cancer Center, Institut de Cancérologie Strasbourg Europe (ICANS), 17 Rue Albert Calmette, BP 23025, 67033 Strasbourg, France; 2 Department of Radiation Oncology, Chongqing University Three Gorges Hospital, 165 Xin Cheng Road, Wanzhou District, Chongqing, 404000 China; 3 Neurosurgery Service, Hautepierre University Hospital, 1, rue Molière, 67000 Strasbourg, France; 4 Statistic Department, UNICANCER, Paul Strauss Comprehensive Cancer Center, Institut de Cancérologie Strasbourg Europe (ICANS), 17 Rue Albert Calmette, BP 23025, 67033 Strasbourg, France; 5 Radiology Department, Amiens-Picardie University Hospital, 1 rond-point du Professeur Christian Cabrol, 80054 Amiens Cedex 1, France

**Keywords:** Hippocampus, Volume, Dose effect, Glioblastoma

## Abstract

**Background:**

The hippocampus is a critical organ for irradiation. Thus, we explored changes in hippocampal volume according to the dose delivered and the location relative to the glioblastoma.

**Methods:**

All patients were treated for glioblastoma with surgery, concomitant radiotherapy and temozolomide, and adjuvant temozolomide. Hippocampi were retrospectively delineated on three MRIs, performed at baseline, at the time of relapse, and on the last MRI available at the end of follow-up. A total of 98, 96, and 82 hippocampi were measured in the 49 patients included in the study, respectively. The patients were stratified into three subgroups according to the dose delivered to 40% of the hippocampus. In the group 1 (n = 6), the hippocampal D_40%_ was < 7.4 Gy, in the group 2 (n = 13), only the H_contra_ D_40%_ was < 7.4 Gy, and in the group 3 (n = 30), the D_40%_ for both hippocampi was > 7.4 Gy.

**Results:**

Regardless of the time of measurement, homolateral hippocampal volumes were significantly lower than those contralateral to the tumor. Regardless of the side, the volumes at the last MRI were significantly lower than those measured at baseline. There was a significant correlation among the decrease in hippocampal volume regardless of its side, and D_max_ (*p* = 0.001), D_98%_ (*p* = 0.028) and D_40%_ (*p* = 0.0002). After adjustment for the time of MRI, these correlations remained significant. According to the D_40%_ and volume at MRI_last_, the hippocampi decreased by 4 mm^3^/Gy overall.

**Conclusions:**

There was a significant relationship between the radiotherapy dose and decrease in hippocampal volume. However, at the lowest doses, the hippocampi seem to exhibit an adaptive increase in their volume, which could indicate a plasticity effect. Consequently, shielding at least one hippocampus by delivering the lowest possible dose is recommended so that cognitive function can be preserved.

*Trial registration* Retrospectively registered.

**Supplementary Information:**

The online version contains supplementary material available at 10.1186/s13014-021-01835-0.

## Introduction

Glioblastoma (GBM), the most common brain cancer in adults, is treated by radiotherapy (RT) plus concurrent and adjuvant temozolomide as first-line treatment in fit patients [1]. Some rare patients can expect an enough longer survival to undergo side-effects of the treatment [2]. Brain RT is well-known to be related with deterioration of neurocognitive functions. New memories were associated with neural stem cells located in the subgranular zone of the hippocampal dentate gyrus [3]. Injury of these cells has been hypothesized to be one of the leading causes of the radiation-induced early cognitive decline [4]. Preclinical studies have shown that low doses of radiotherapy are sufficient to induce a decrease in neurogenesis in the subgranular zone. This loss in neurogenic capacity is reportedly correlated with a decline in new memory formation and impaired recall [5]. Furthermore, clinical trials have demonstrated the validity of these preclinical results by dosimetric analysis [6, 7]. Fortunately, new radiotherapy techniques, such intensity-modulated radiation therapy (IMRT), have helped to protect hippocampi and prevent cognitive decline [8–11].

According to trials investigating brain metastasis, dose constraints have been described for both hippocampi [7, 10]. In the setting of partial-brain irradiation, there has also been evidence indicating that a higher radiotherapy (RT) dose to the hippocampus may be associated with greater memory impairment [6, 12, 13]. Ali et al. demonstrated that redefining the CTV for the GBMs led to decrease dramatically dose delivered to the hippocampi [14].

Trials studying hippocampal shielding and cognitive consequences have mainly been designed for and conducted in patients with whole-brain irradiation or stereotactic irradiation of multiple metastases leading to an equivalent dose in both hippocampi [7, 9, 10]. In glioma, the radiation fields were mainly asymmetrical, leading to a significant delivered dose and allowing for one hippocampus to be shielded [15]. Wee et al. showing that this hippocampi protection did not increase the risk of GBM relapse [16]

Physiologically, the decreasing hippocampi volume in one year was between 0.8 and 4.4% of the initial volume [17]. In a meta-analysis, the hippocampus atrophy rates in the both hippocampi, the left hippocampus, and the right hippocampus were 0.85%, 0.64%, and 0.70%, respectively. For both hippocampi, the atrophy rate differed according to the age and was 0.38%, 0.98%, and 1.12% in patients younger than 50, between 50 and 70, and older than 70, respectively [18]. Hippocampal volume tracking with structural MRI has proven clinical utility in a variety of diseases, including Alzheimer's disease [19, 20], temporal lobe epilepsy [21], and traumatic brain injury [22]. Interestingly, Maguire et al. showed that taxi drivers' hippocampi were larger than those of other people, which was not correlated with innate navigational expertise but with training and their ability to use their spatial knowledge [23, 24].

Many authors have investigated the role of hippocampal and memory disorders in numerous pathologies [12, 21, 25–29]. Thus, complementary studies on the consequences of hippocampal irradiation are warranted to improve memory preservation in brain radiotherapy patients [30].

tablThe first purpose of the study was to evaluate changes in hippocampus size among irradiated GBM patients during the follow-up according to the tumor side and the received dose. Secondly, this study tried to assess whether the changes to the nonirradiated/low-irradiated hippocampus are similar to those of the higher-irradiated hippocampus.

## Methods

The institutional review board approved this retrospective study. All patients gave their consent to collect and analyze their data, and all live patients specifically agreed to participate in this study, according to the French CNIL law MR004.

Forty-nine patients with GBM, treated with irradiation, were retrospectively analyzed in this study. There were 34 males and 15 females with a median age of 61-years old (mean: 60.6; min–max: 24–81). Twenty-five tumors were located in the left cerebral hemisphere, and 24 were located in the right cerebral hemisphere.

### Imaging acquisition

All MR images were acquired using a Signa Excite HDx 3.T™ system (GE Healthcare, Milwaukee, WI) with an 8-channel dedicated head coil. The MRI scanning protocol included pre- and postcontrast 1-mm, 3-dimensional (3D) volumetric T1-weighted multiecho magnetization-prepared rapid-acquisition gradient echo (MPRAGE) images, and 3D T2-weighted fluid-attenuated inversion recovery (FLAIR) images. Three MRI image sets were analyzed. The first MRI was obtained with a median interval of 13 days (mean 12,7; min–max: 5–23) before the start of RT (MRI_dosimetric_), the second at the time of relapse (MRI_relapse_), with a median interval of 4.6 months (mean 7.2; min–max: 1.1–22.0) after the end of RT, and the third was the last MRI during follow-up (MRI_last_), with a median interval of 17.6 months (mean 17.7; min–max: 3.3–44.3) after the end of RT. The median time interval between MRI_relapse_ and MRI_last_ was 11.4 months (mean 13;1; 1.9–36.7).

The Planning Target Volume (PTV) included tumors visualized on a gadolinium-enhanced T1 weighted MPRAGE sequence plus a 10 mm-margin completed with edema in the FLAIR sequence, finally encompassed by a 3-mm margin. GBM patients were irradiated at a dose of 60 Gy in 30 daily fractions of 2 Gy, five days a week. All the patients received concomitant chemotherapy with temozolomide at a median daily dose of 140 mg (mean 135.85; min–max: 120–160). Forty-three patients underwent a median number of 6 cycles (1–10) of adjuvant chemotherapy at a median daily dose of 340 mg (mean 330; 140–400) according to the EORTC/NCIC protocol [31].

### Hippocampus delineation

Hippocampi were prospectively delineated on the gadolinium-enhanced T1-weighted MPRAGE sequence with 1-mm slices MRI_dosimetric_ and retrospectively delineated from the same MRI sequence on the MRI_relapse,_ and the MRI_last_, according to atlas [8, 32]. Hippocampal delineation was performed by a radiation oncologist with a five years of experience (XC) and approved by a radiation oncologist (GN) with over 20 years of experience [33].

Hippocampi were not included if there was any distortion in the hippocampal anatomy due to postsurgical effects or proximity/invasion of the tumor. Hippocampal volumes were stratified into contralateral (H_contra_) and homolateral (H_homo_) to the GBM, and the composite bilateral consisted of the sum of H_contra_ and H_homo_ (H_sum_). At baseline MRI_dosimetric_, MRI_relapse,_ and MRI_last_, the numbers of delineated hippocampi were 98, 96, and 82, respectively. No patient had both hippocampi censored.

### Scheduled doses to hippocampi

The dose constraint was D_40%_ < 7.4 Gy for H_sum_. If this aim could not be reached (mainly due to the proximity of one hippocampus to the tumor), then this constraint was imposed on the contralateral hippocampus. In the case of cross-median line GBM, the planning tried to reach the lowest dose as possible in the H_contra_. However, hippocampal constraint respect was never preferred to the tumor coverage (D98% > 95% of the prescribed dose) to limit the risk of GBM relapse.

Finally, the entire patient group was split into three subgroups according to the dose delivered to 40% of the hippocampus. In group 1 (n = 6), the hippocampal D_40%_ was < 7.4 Gy, in group 2 (n = 13), only H_contra_ D_40%_ was < 7.4 Gy, and in group 3 (n = 30), both hippocampal D_40%_ were > 7.4 Gy. Furthermore, hippocampi were split into four subgroups according to the D_40%_, < 7.4 Gy, between 7.4 Gy and < 30 Gy, between ≥ 30 Gy and < 50 Gy, and ≥ 50 Gy.

## Statistics

Volumes of hippocampi were determined on MRI_dosimetric_ (H_homo-j0_, H_contra-j0,_ and H_sum-j0_), on MRI_relapse_ (H_homo-relapse_, H_contra-relapse,_ and H_sum-relapse_), and MRI_last_ (H_homo-last_, H_contra-last,_ and H_sum-last_).

The minimum dose (D_min_), mean dose ($\stackrel{-}{D}$), maximum dose (D_max_), D_2%_, D_10%_, D_40%_, D_50%_, D_80%,_ and D_98%_ were collected for each hippocampus and for the combination of both. According to the linear-quadratic model, for the hippocampi receiving less than 2 Gy per fraction, doses were recalculated with an α/β = 2 Gy. The change in hippocampal volumes was analyzed according to the doses, follow-up time, and contact/proximity to the GBM using Pearson's product-moment correlation. Comparisons of the distribution of volumes, doses, and percentages between homolateral and contralateral hippocampus were performed with the T.Test. RStudio Version 1.2.5033 was used to perform statistical calculations.

## Results

### Hippocampal volumes and time of measure

#### Overall patients

The volumes are presented in Table 1. Regardless of the time of measurement, the volume of H_homo_ was always significantly lower than those of H_contra_, H_homo-j0_ versus H_contra-j0_ (*p* = 0.02), H_homo-relapse_ versus H_contra-relapse_ (*p* < 0.002), and H_homo-last_ versus H_contra-last_ (*p* < 0.003) (Additional file 1: Annex 1). Regardless of the side, the volume at the last measurement was always significantly lower than that measured at baseline, H_homo-j0_ versus H_homo-last_ (*p* = 0.02), H_contra-j0_ versus H_contra-last_ (*p* = 0.049). There was no significant difference in the measurements between MRI_relapse_ and MRI_last_, neither for H_homo_ nor H_contra_ (Additional file 1: Annex 1a).

**Table 1:** Hippocampi volume, change in volume and percent change according to the interval between MRIs

	H_homo_	H_contra_	H_sum_
	Median volume (min–max) mm^3^		
MRI_dosimetric_	3400 (650–4850)	3540 (2000–4680)	6940 (3600–9530)
MRI_relapse_	3150 (610–4630)	3410 (2030–4440)	6480 (3080–9050)
MRI_last_	3060 (400–4230)	3350 (1860–5780)	6340 (3240–8290)
	Median reduction between MRI_dosimetric_ and MRI_relapse_		
Volume (mm^3^)	− 310 (+ 840 to − 2750)	− 140 (+ 500 to − 1160)	− 380 (+ 1170 to − 3460)
%	− 9.5 (+ 36.0 to − 80.9)	− 4.0 (+ 15.9 to − 32.0)	− 5.3 (+ 17.9 to − 87.6)
	Median reduction between MRI_dosimetric_ and MRI_last_		
Volume (mm^3^)	− 520 (+ 500 to − 1157)	− 190 (+ 1720 to − 201)	− 720 (+ 1200 to − 2310)
%	− 17.6 (+ 14.7 to − 61.8)	− 5.4 (+ 42.4 to − 50.1)	− 10.3 (+ 18.3 to − 35.8)
	Median reduction between MRI_relapse_ and MRI_last_		
Volume (mm^3^)	− 150 (+ 960 to − 1470)	− 190 (+ 1390 to − 1370)	− 290 (+ 1400 to − 2230)
%	− 5.0 (+ 25.9 to − 151.5)	− 5.7 (+ 32.2 to − 73.7)	− 4.2 (+ 18.1 to − 57.0)

#### Group stratification

The volumes are presented in Table 2. According to intragroup comparisons, only for group 3 was the volume of H_homo-G3_ always lower than those of H_contra-G3_, H_homo-j0-G3_ versus H_contra-j0-G3_ (*p* = 0.01), H_homo-relapse-G3_ versus H_contra-relapse-G3_ (*p* = 0.01), and H_homo-last-G3_ versus H_contra-last-G3_ (*p* = 0.01) (Additional file 2: Annex 2a).

**Table 2:** Hippocampi volume and change in volume between MRIs according to the D40% groups

	H_homo-G1_	H_contra-G1_	H_homo-G2_	H_contra-G2_	H_homo-G3_	H_contra-G3_
	Median volume (min–max) (mm^3^)					
MRI_dosimetric_	3700 (3070–4410)	3460 (2880–4010)	3540 (2330–4850)	3450 (2180–4680)	3250 (650–4320)	3640 (2000–4350)
MRI_relapse_	3260 (3120–4390)	3340 (2760–4070)	3050 (1960–4630)	3380 (2340–4440)	3030 (610–3970)	3500 (2030–4120)
MRI_last_	3200 (3020–3560)	3350 (3200–3620)	2670 (1970–4230)	3440 (1860–5780)	3080 (400–3620)	3310 (2000–4350)
	Median reduction between MRI_dosimetric_ and MRI_relapse_					
Volume (mm^3^)	− 200 (+ 180 to − 1140)	− 70 (+ 220 to − 590)	− 420 (+ 840 to − 950)	− 100 (+ 330 to − 690)	− 220 (+ 580 to − 2750)	− 220 (+ 500 to − 1160)
%	− 5.4 (+ 5.5 to − 26.4)	− 1.9 (+ 7.6 to − 17.6)	− 10.6 (+ 36 to − 25.9)	− 3.1 (+ 10.8 to − 17.8)	− 8.7 (+ 21.5 to − 80.9)	− 5.3 (+ 15.9 to − 32.0)
	Median reduction between MRI_relapse_ and MRI_last_					
Volume (mm^3^)	− 60 (+ 250 to − 1370)	+ 10 (+ 100 to − 500)	− 150 (+ 960 to − 970)	− 20 (+ 1390 to − 1370)	− 270 (+ 500 to − 1470)	− 210 (+ 1170 to − 1300)
%	− 1.7 (+ 7.5 to − 31.2)	+ 0.3 (+ 3.2 to − 13.3)	− 4.8 (+ 35 to − 29.5)	− 0.6 (+ 31.7 to − 42.4)	− 12.4 (+ 16.0 to − 60.2)	− 6.9 (+ 47.6 to − 39.4)
	Median reduction between MRI_dosimetric_ and MRI_last_					
Volume (mm^3^)	− 130 (+ 130 to − 1390)	− 180 (+ 320 to − 600)	− 620 (+ 500 to − 1400)	− 190 (+ 1720 to − 1230)	− 500 (+ 150 to − 1570)	− 190 (+ 840 to − 2010)
%	− 3.5 (+ 4.2 to − 31.5)	− 5.1 (+ 11.1 to − 15.5)	− 20.9 (+ 14.7 to − 38.1)	− 5.6 (+ 42.4 to − 39.8)	− 17.6 (+ 5 to − 61.8)	− 5.4 (+ 23.9 to − 50.1)

In group 1 (G1: n = 6), in both hippocampi, the D_40%_ was < 7.4 Gy; in group 2 (G2: n = 13), the H_contra_ D_40%_ was < 7.4 Gy; and in group 3 (G3: n = 30), the D_40%_ for both hippocampi was > 7.4 Gy

According to intergroup analysis, significant decreases in volume were observed between G1 and G3 for H_homo-j0-G1_ versus H_homo-j0-G3_ (*p* = 0.03), H_homo-relapse-G1_ versus H_homo-relapse-G3_ (*p* = 0.02), H_homo-last-G1_ versus H_homo-last-G3_ (*p* = 0.01) and H_contra-last-G1_ versus H_contra-last-G3_ (*p* < 0.01). There was no significant difference in volume between G1 and G2 and between G2 and G3 (Additional file 2: Annex 2a).

### Volume differences between MRI_dosimetric_ and MRI_relapse_

#### Overall patients (Table 1)

For H_homo_, the median volume of reduction was − 310 mm^3^ corresponding to a difference of − 9.5%, (*p* = 0.02 and *p* = 0.02, respectively) (Additional file 1: Annex 1b). For H_contra_, the median volume of reduction was − 140 mm^3^ corresponding to a difference of − 3.97% (*p* = 0.02 and *p* = 0.02, respectively) (Additional file 1: Annex 1b).

#### Group stratification (Table 2)

According to intra- or inter-group analysis, no significant differences were observed (Additional file 2: Annex 2b, 2c).

### Volume differences between MRI_dosimetric_ and MRI_last_

#### Overall patients (Table 1)

For H_homo_, the median volume of reduction was − 520 mm^3^, representing a difference of − 17.6% (*p* = 0.03 and *p* = 0.01, respectively) (Additional file 1: Annex 1b). For H_contra_, the median volume of reduction was − 190 mm^3^, representing a difference of − 5.37% (*p* = 0.03 and *p* = 0.01, respectively) (Additional file 1: Annex 1b).

#### Group stratification (Table 2)

According to intragroup analysis, differences were only significant for H_contra-dosi-G3_ versus H_contra-last-G3_, and their median volumes were 3640 mm^3^ and 3310 mm^3^ (*p* = 0.18) (Additional file 2: Annex 2b), representing a difference of − 5.37% (*p* = 0.03) (Additional file 2: Annex 2c). According to intergroup analysis, no significant difference was observed.

### Volume difference between MRI_relapse_ and MRI_last_

#### Overall patients (Table 1)

For H_homo_ and H_contra_, volume reduction was not significantly different (Additional file 1: Annex 1b).

#### Group stratification (Table 2)

According to intra- or intergroup analysis, no significant differences were observed (Additional file 2: Annex 2b, 2c).

### Dose distribution and volume

#### Overall patients (Table 3a)

**Table 3:** Median dose in the hippocampi

		Dmean	Dmax	D10%	D20%	D30%	D40%	D50%	D60%	D70%	D80%	D90%	D100%
(a)													
Homolateral hippocampus	Min	1.30	1.86	1.51	1.40	1.34	1.30	1.27	1.24	1.20	1.16	1.12	1.03
	Max	61.12	63.49	62.82	62.16	61.51	61.35	61.18	60.97	60.75	60.53	60.25	59.65
	median	53.85	59.99	59.37	59.10	58.74	57.94	56.63	53.93	50.87	45.02	38.21	35.33
Contralateral hippocampus	Min	1.26	1.99	1.52	1.37	1.31	1.27	1.23	1.10	0.94	0.83	0.73	0.58
	Max	46.88	61.07	58.33	56.23	52.73	49.68	46.27	42.65	40.35	38.34	37.62	36.71
	median	13.89	33.26	20.41	16.42	14.57	11.50	11.15	10.87	10.74	10.60	9.72	3.80

			Dmean	Dmax	D10%	D20%	D30%	D40%	D50%	D60%	D70%	D80%	D90%	D100%
(b)														
Group 1	Homolateral hippocampus	Min	1.30	1.86	1.51	1.40	1.34	1.30	1.27	1.24	1.20	1.16	1.12	1.03
		Max	5.06	17.63	8.47	7.39	6.02	5.06	4.41	3.91	3.58	3.27	2.98	1.71
		median	2.78	6.32	4.77	3.69	2.64	2.30	2.19	2.04	1.94	1.80	1.71	1.35
	Contralateral hippocampus	Min	1.26	1.99	1.52	1.37	1.31	1.27	1.23	1.19	1.11	1.00	0.90	0.77
		Max	4.80	13.12	6.97	5.77	5.20	4.74	4.36	4.08	3.81	3.52	3.22	1.05
		median	1.99	5.47	3.50	2.11	1.73	1.60	1.47	1.38	1.30	1.20	1.12	0.99
Group 2	Homolateral hippocampus	Min	8.44	17.94	10.78	10.65	10.35	8.56	7.87	7.87	4.12	3.27	2.44	4.79
		Max	59.96	61.83	61.08	60.93	60.78	60.56	60.32	60.32	59.72	59.22	58.88	54.03
		median	35.54	54.95	45.74	43.05	40.98	38.47	34.90	34.90	22.06	17.04	15.34	7.50
	Contralateral hippocampus	Min	3.28	11.30	5.31	3.56	3.15	2.25	1.31	1.10	0.94	0.83	0.73	0.58
		Max	12.59	54.15	34.18	25.58	13.73	7.40	6.22	5.94	5.51	4.91	4.16	3.57
		median	6.16	21.06	10.17	7.43	6.59	5.14	4.70	3.30	3.11	2.94	2.75	2.46
Group 3	Homolateral hippocampus	Min	17.60	25.00	21.30	20.01	19.13	18.17	17.35	16.52	15.75	14.70	9.26	9.25
		Max	61.12	63.49	62.82	62.16	61.51	61.35	61.18	60.97	60.75	60.53	60.25	59.65
		median	57.87	60.77	60.18	59.72	59.31	59.25	58.98	58.73	57.98	56.79	55.49	44.26
	Contralateral hippocampus	Min	8.17	16.02	11.76	11.42	10.64	8.12	5.86	4.61	3.63	2.80	2.21	2.31
		Max	46.88	61.07	58.33	56.23	52.73	49.68	46.27	42.65	40.35	38.34	37.62	36.71
		median	19.57	38.32	29.41	23.58	19.40	18.52	17.95	17.31	16.08	15.47	14.53	13.96

On both sides, the volume decrease at MRI_last_ time was correlated with D_max_, D_98%_ and D_40%_ (*p* = 0.0011, *p* < 0.001 and *p* = 0.0002, respectively).

For D_min_, D_2%_, D_max_, D_98%_, $\stackrel{-}{D},$ D_10%_, D_40%_, D_50%_, D_80%_, and D_100%_, the values for H_homo_ were significantly higher than those for H_contra_ (*p* < 0.0001 for all comparisons).

Before and after recalculation with a 2-Gy equivalent-dose, each analyzed dose value was significantly higher for H_homo_ than for H_contra_ (*p* < 0.0001 for all comparisons).

#### Group stratification (Table 3b)

D_40%_ and D_40%Eq. 2 Gy_ were studied among the three groups. For group 1, there was no difference in D_40%_ and D_40%Eq. 2 Gy_ for H_homo_ and H_contra_. For group 2, the median D_40%_ and D_40%Eq. 2 Gy_ values were significantly higher in H_homo_ than in H_contra,_ 38.5 Gy versus 5.1 Gy (*p* < 0.001) and 31.6 Gy and 2.8 Gy (*p* < 0.0001), respectively. For group 3, comparable differences were observed for 59.3 Gy versus 18.5 Gy (*p* < 0.001) and 58.9 Gy versus 12.1 Gy (*p* < 0.0001), respectively.

### Correlation between hippocampus volumes and dose

#### Overall patients (Table 4)

**Table 4:** Volume size changes according to hippocampi groups

	# of patients	Homolateral		Contralateral	
		VL (mm^3^/Gy)	%L (%/Gy)	VL (mm^3^/Gy)	%L (%/Gy)
(a)					
Group 1	6	− 124	+ 1.5	+ 172	+ 4.0
Group 2	13	− 15	− 0.51	+ 15	+ 4.3
Group 3	30	− 19.7	− 0.04	− 19.7	− 0.52

	D40% ≤ 7.4 Gy	7.4 Gy < D40% ≤ 30 Gy	30 Gy < D40% ≤ 50 Gy	D40% > 50 Gy
(b)				
Slope VL	+ 94.3 mm^3^/Gy	− 8.6 mm^3^/Gy	− 45.4 mm^3^/Gy	− 112.2 mm^3^/Gy
Slope %L	+ 2.7 %/Gy	− 0.44 %/Gy	− 1.13 %/Gy	− 5.55 %/Gy

(a) Slope values of the volume lost (VL, mm^3^/Gy) or percentage of volume lost (L − %/Gy) in each hippocampus (homo or contralateral to the tumor) and for the three groups stratified by D40% in both hippocampi: In group 1 (G1: n = 6), in both hippocampi, the D_40%_ was < 7.4 Gy; in group 2 (G2: n = 13), the H_contra_ D_40%_ was < 7.4 Gy; in group 3 (G3: n = 30), the D_40%_ for both hippocampi was > 7.4 Gy. (b) Slope values of volume lost: The volume lost (VL) or percentage of volume lost (%L − %/Gy) according to the dose for both hippocampi between the reference MRI (dosimetric MRI) and last MRI during follow-up

There was a significant correlation between the decrease in the volume of the hippocampus, regardless of its side and D_max_ (*p* = 0.001), D_98%_ (*p* = 0.028) and D_40%_ (*p* = 0.0002). Adjusted to the time of analysis, these correlations remained significant. According to D_40%_ and volume at MRI_last_ time, overall hippocampi decreased by 4 mm^3^/Gy. However, these changes were not linear when the doses were stratified into four subgroups, < 7.4 Gy, between 7.4 Gy and < 30 Gy, between ≥ 30 Gy and < 50 Gy, and ≥ 50 Gy. The slopes were + 94.3 mm^3^/Gy, − 8.6 mm^3^/Gy, − 44.5 mm^3^/Gy, and − 112.2 mm^3^/Gy, respectively.

#### Group stratification (Table 4)

For group 1, the change in volume for H_homo_ and H_contra_ from MRI_dosimetric_ to MRI_last_, according to D_40%_, was opposite, with slopes of − 124 mm^3^/Gy and + 172 mm^3^/Gy, respectively.

For group 2, H_homo_ and Hcontra's evolution was also opposite, − 15 mm^3^/Gy and + 154 mm^3^/Gy, respectively.

For group 3, the slopes of the change in volume for H_homo_ and H_contra_ volumes followed the same directions, with − 19.7 mm^3^/Gy and − 19.7 mm^3^/Gy, respectively.

## Discussion

The dose constraints of hippocampi are currently well defined to dramatically and efficiently decrease the hippocampal dose and, consequently, memory impairment. However, these dose constraints were primarily referenced by D_40%_, including both hippocampi, and were proposed secondary to the results of the first study, which used whole-brain radiation therapy, where hippocampi were irradiated or shielded symmetrically. In contrast, only two studies have focused on asymmetric irradiation in glioma [6, 34].

To our knowledge, this is the first study to investigate D_40%_ in hippocampal volumes measured by MRI and to analyze the change in the hippocampus contralateral to the GBM after irradiation. This study clearly showed that the volume of hippocampi decreased after radiotherapy in patients irradiated for GBM. However, the decrease in hippocampal size depended on the tumor side and relied on the received radiation dose. These factors could explain the variability in memory disturbances after brain irradiation.

Delineation of hippocampi, which requires training and support of the atlas, have been recommended [8, 32]. In the study by Gondi et al*.*, for protection, hippocampi were manually delineated according to the protocol but only after the planning dose calculation was determined [6]. Notably, Siebert et al. used an automated segmentation method that is more reproducible than manual tracing that requires more expertise and training. Furthermore, in the Siebert et al. studies, all images were obtained with the same MRI devices, which required conditions to optimize the automated delineation that often deviated from daily practice [35–37]. Computerized segmentation volume methods were shown to be competitive with expert segmentation [25]. The main advantage of automated processes is the decrease in interobserver variability. However, automatic segmentation methods have enabled the subevaluation of hippocampal atrophy that develops over time [38]. In the present study, only one radiation oncologist delineated all the hippocampi, which improved the quality of volume comparison and removed the interobserver variability.

In the current series, the median decrease in hippocampal volumes varied from 4 to 17.6% depending on the tumor side, received dose, and time after irradiation. In contrast, Prust et al. did not observe any change in the nine-month MRI-follow-up in 14 patients treated for GBM [39]. This difference of change is likely the consequence of the longer MRI follow-up in the current study, at 17.6 months between the first and the last MRI.

Gondi et al. did not show any correlation with the hippocampus analyzed separately [6]. In contrast, the current study showed that the hippocampal volume decrease is dependent on location of the hippocampus relative to the tumor. At the time of last MRI, the percent decrease in volume was more substantial in the homolateral hippocampus than in the contralateral hippocampus, at 17.6% and 5.4%, respectively. We demonstrated a clear relationship between the post-irradiation time and hippocampal atrophy, with substantial changes appearing in the first months after RT.

In this work, we showed that the median volume of the homolateral hippocampi relative to the glioblastoma was always lower that of the contralateral hippocampi. The impact of glioblastoma on hippocampi functioning and homeostasis is unknown, but these results suggest an interaction. However, the consequences of surgery always being performed before the reference MRI (MRI_dosimetry_) cannot be excluded, but other causes should also be considered (medicine, age, addiction, estrogen level, corticosteroid intake…) [40]. Another assumed reason to explain this difference is the possible ability of the contralateral hippocampus to compensate for the decrease in the homolateral hippocampus volume after a low dose of irradiation, as a plasticity effect has already been shown in some variable situations [26, 28, 41].

Animal studies have shown that when the brains of young rats are unilaterally irradiated, the volume of the irradiated hippocampus is reduced compared to that of the nonirradiated side, corresponding to apoptosis, which induces the loss of neural stem cells and progenitor cell proliferation [42, 43]. A postmortem study on patients treated with chemotherapy and cranial irradiation showed profoundly reduced hippocampal neurogenesis. This observation further supports the hypothesis that neurocognitive impairment after brain-directed therapy hampers hippocampal neurogenesis to some degree [44, 45].

In the study by Gondi et al., risk impairment was significantly correlated with a D_40%_ in the bilateral hippocampi > 7.4 Gy (*p* = 0.043) [6].

Seibert et al. showed that hippocampal volume loss was significantly correlated with the mean RT dose delivered to the hippocampus (*p* = 0.03). The mean hippocampal volume was significantly reduced one year after high-dose (> 40 Gy) radiation therapy, but not after low-dose (< 10 Gy) radiation therapy [34]. In the current study, there was a correlation between the delivered D_max_, D_98%,_ and D_40%_ with decreasing hippocampal volume. Furthermore, we showed that the volume decreased continuously with D_40%_ from > 7.5 to > 50 Gy. Notably, for a D_40%_ < 7.4 Gy, hippocampal volumes increased. Dose-dependent brain changes were also demonstrated for white matter [46], the amygdala [47], and left-sided perisylvian white matter [48]. In our study, the hippocampi receiving less than 7.4 Gy were always contralateral hippocampus relative to the GBM, and in 7 cases, the homolateral hippocampus whom D_40%_ was < 7.4 Gy because the GBM was far enough away from the hippocampus and consequently, the hippocampus was not in, or near the radiation fields.

Siebert et al. showed a one-year atrophy rate of 6% in the hippocampi that received a dose > 40 Gy [34]. This value is comparable to the 1% volume loss per year observed in the elderly [17, 18] and the 2.2 to 4% volume loss per year observed in Alzheimer’s patients with mild to severe cognitive decline [17, 38, 49]. For the entire series, we noted a median decrease of 0.33% in hippocampal volumes over a median period of 17.5 months (time between MRI_dosimetry_ and MRI_last_), but a median reduction of 5.55% in hippocampi that received more than 50 Gy in the same period.

In our current series, contralateral hippocampi that received a D_40%_ less than 7.4 Gy did not show any atrophy in the hippocampus; in contrast the volume increased significantly. The lack of hippocampus atrophy at low dose was reported in several previous study [7, 34, 36, 39]. Physiological and functional compensations could explain these observations, but methods to specifically study each hippocampus separately have not yet been developed. At present, our study cannot confirm that when the dose of irradiation was low, the increased volume was an adaptive reaction to irradiation, a natural adaptation to the brain trauma or functional adaptation to compensate memory ability loss. Interestingly, Ericksonn et al. showed that physical activity training increased hippocampal perfusion, reversing effect of age-related loss [50]. Memory training can also increase the hippocampi volumes as showed studies in taxi drivers [23, 24].

Notably, regardless of the tumor distance, the homolateral hippocampus volume was always significantly smaller than the contralateral hippocampus volume. This relative atrophy suggested that dose was not the sole cause of this decline. Other causes could be vascular disruption and permeability [29], alteration of interneurons [27], and neuroinflammation [46]. This difference in hippocampal volume has already been described in hippocampal sclerosis and epilepsy [17, 21, 25, 38]. However, it is unknown whatever this difference in volume was due to a variation secondary to atrophy alone (i.e., the contralateral hippocampus having a normal volume) or atrophy and unaltered volume compensation in the contralateral hippocampus [33].

This study was limited by the absence of specific cognitive performance measures to correlate with the observed structural neuroimaging changes. Validated cognitive tests are not always used in routine clinical practice, precluding clinical neurologic observation analysis in retrospective studies. However, these tests should be precise and split the left or right hippocampus [51], and dose thresholds should be relevantly chosen [52] to avoid unclear or confusing analysis. Moreover, advanced imaging access is still limited in medical practice, and other brain regions are involved in cognitive functions [46, 53]. Another drawback is the lack of a control group to measure the change in the hippocampus over time in a population based on age, IK,…. However, this requirement could be disputed because of the absence of tumors, which probably interact with the hippocampal structure through the microenvironment.

This study showed that the low-irradiated hippocampus volume and/or far from the GBM changes differently than the hippocampus near to the tumor and/or receiving high irradiated doses. These observations suggest that tumor could more deteriorate hippocampus structure and function than radiotherapy and that shielding of H_contra_ could give possibility of hippocampus volume adaptation and function recovery. However, several future improvement have to be directed to demonstrate the clinical hypotheses [30]. The role of protontherapy should be more extensively compared to optimal modulated photon radiation [54]. The volume of protection, total or partial hippocampus, with or without 1-cm margin should be more specify. Because, physical doses can be highly different according to the delivered dose, the dose distribution and the dose per fraction protocol, a better knowledge of biological dose and of the hippocampal α/β value is required [30]. Ultimately, maybe not all patients need a hippocampal protection, a better and more systematic neurocognitive function initial evaluation is required to select the best candidates for useful protection. This evaluation requires standardized tools, able to measure early and late changes, which are likely not be the equal, as well as right and left hippocampal functions probably different [55]. In addition, the testing must be feasible to administer in a busy clinical practice. Finally, cognitive training could improve function, mainly of the shielded hippocampus [50]

To correlate neurocognitive outcomes with structural brain changes, prospective longitudinal trials are needed to examine performance in multiple cognitive domains in concert with serial neuroimaging [56].

## Conclusion

This study demonstrated a D_40%_-dependent atrophy effect on the irradiated hippocampus. The volume of the contralateral hippocampus increased when irradiated at a D_40%_ < 7.4 Gy increased, suggesting a compensatory reaction. Thus, limiting the radiation dose to the greatest extent possible in at least one hippocampus is recommended, when relevant, in cases of asymmetrical brain cancer.

## Supplementary Information

**Additional file 1**. Annex 1: Relevant comparison of volumes according to MRI. Annex 1a: Comparison of volumes measured on MRI. Annex 1b: Comparison between changes in volume during the intervals between MRIs. Annex 1c: Comparison between changes in percent volume during the intervals between MRIs

**Additional file 2**. Annex 2: Relevant comparison of hippocampal volumes their changes according to the time of the MRIs. Annex 2a: Comparison between volumes at a given MRI time according to subgroup: In group 1 (G1: n=6), in both hippocampi, the D_40%_ was < 7.4 Gy; in group 2 (G2: n=13), the H_contra_ D_40%_ was < 7.4 Gy; and in group 3 (G3: n=30), the D_40%_ for both hippocampi was > 7.4 Gy. Annex 2b: Comparison between volumes measured on different MRIs and according to subgroup: In group 1 (G1: n=6), in both hippocampi, the D_40%_ was < 7.4 Gy; in group 2 (G2: n=13), the H_contra_ D_40%_ was < 7.4 Gy; and in group 3 (G3: n=30), the D_40%_ for both hippocampi was > 7.4 Gy. Annex 2c: Comparison between changes in volumes during the interval between MRIs and according to subgroup: In group 1 (G1: n=6), in both hippocampi, the D_40%_ was < 7.4 Gy; in group 2 (G2: n=13), the H_contra_ D_40%_ was < 7.4 Gy; and in group 3 (G3: n=30), the D_40%_ for both hippocampi was > 7.4 Gy. Annex 2d: Comparison between changes in % volumes during interval between MRIs and according to subgroup: In group 1 (G1: n=6), in both hippocampi, the D_40%_ was < 7.4 Gy; in group 2 (G2: n=13), the H_contra_ D_40%_ was < 7.4 Gy; and in group 3 (G3: n=30), the D_40%_ for both hippocampi was > 7.4 Gy

## Data Availability

The datasets used and/or analyzed during the current study are available from the corresponding author on reasonable request.

## Abbreviations

FLAIR: Fluid-attenuated inversion recovery
GBM: Glioblastoma
IMRT: Intensity-modulated radiation therapy
MPRAGE: Multiecho magnetization-prepared rapid-acquisition gradient echo
PTV: Planning target volume
RT: Radiotherapy
