# Supplementary material for: Role of hippocampal location and radiation dose in glioblastoma patients with hippocampal atrophy
Source: Radiat Oncol. 2021 Jun 22;16:112. doi: 10.1186/s13014-021-01835-0 (PMC8220779; doi:10.1186/s13014-021-01835-0)
Supplement: Supplementary file 1 — Additional file 1. Annex 1: Relevant comparison of volumes according to MRI. Annex 1a: Comparison of volumes measured on MRI. Annex 1b: Comparison between changes in volume during the intervals between MRIs. Annex 1c: Comparison between changes in percent volume during the intervals between MRIs [file 13014_2021_1835_MOESM1_ESM.docx]

Annex 1: Relevant comparison of volumes according to MRI

Annex 1a: Comparison of volumes measured on MRI

| *p* | H_homo-_MRI_dosimetry_ | H_contra-_MRI_dosimetry_ | H_homo-_ MRI_relapse_ | H_contra-_ MRI_relapse_ | H_homo-_ MRI_last_ | H_contra-_ MRI_last_ |
| --- | --- | --- | --- | --- | --- | --- |
| H_homo-_ MRI_dosimetry_ |  | 0.02 | 0.02 |  | 0.02 |  |
| H_contra-_ MRI_dosimetry_ | 0.02 |  |  | 0.17 |  | 0.049 |
| H_homo-_MRI_relapse_ | 0.02 |  |  | 0.002 | 0.53 |  |
| H_contra-_MRI_relapse_ |  | 0.17 | 0.002 |  |  | 0.41 |
| H_homo-_MRI_last_ | 0.02 |  | 0.53 |  |  | 0.003 |
| H_contra-_MRI_last_ |  | 0.049 |  | 0.41 | 0.003 |  |

Annex 1b: Comparison between changes in volume during the intervals between MRIs

| Volumes changes between MRI_dosimetric_ and MRI_relapse_ | | | |
| --- | --- | --- | --- |
| *p* | H_homo_ | H_contra_ |  |
| H_homo_ |  | 0.02 |  |
| H_contra_ | 0.02 |  |  |

| Volumes changes between MRI_relapse_ and MRI_last_ | | | |
| --- | --- | --- | --- |
| *p* | H_homo_ | H_contra_ |  |
| H_homo_ |  | 0.19 |  |
| H_contra_ | 0.19 |  |  |

| Volumes changes between MRI_dosimetry_ and MRI_last_ | | | |
| --- | --- | --- | --- |
| *p* | H_homo_ | H_contra_ |  |
| H_homo_ |  | 0.03 |  |
| H_contra_ | 0.03 |  |  |

Annex 1c: Comparison between changes in percent volume during the intervals between MRIs

| % of volumes changes between MRI_dosimetric_ and MRI_relapse_ | | |
| --- | --- | --- |
| *p* | H_homo_ | H_contra_ |
| H_homo_ |  | 0.02 |
| H_contra_ | 0.02 |  |

| % of volumes changes between MRI_relapse_ and MRI_last_ | | |
| --- | --- | --- |
| *p* | H_homo_ | H_contra_ |
| H_homo_ |  | 0.10 |
| H_contra_ | 0.10 |  |

| % of volumes changes between MRI_relapse_ and MRI_last_ | | |
| --- | --- | --- |
| *p* | H_homo_ | H_contra_ |
| H_homo_ |  | 0.01 |
| H_contra_ | 0.01 |  |
