# Supplementary material for: Role of hippocampal location and radiation dose in glioblastoma patients with hippocampal atrophy
Source: Radiat Oncol. 2021 Jun 22;16:112. doi: 10.1186/s13014-021-01835-0 (PMC8220779; doi:10.1186/s13014-021-01835-0)
Supplement: Supplementary file 2 — Additional file 2. Annex 2: Relevant comparison of hippocampal volumes their changes according to the time of the MRIs. Annex 2a: Comparison between volumes at a given MRI time according to subgroup: In group 1 (G1: n=6), in both hippocampi, the D40% was < 7.4 Gy; in group 2 (G2: n=13), the Hcontra D40% was < 7.4 Gy; and in group 3 (G3: n=30), the D40% for both hippocampi was > 7.4 Gy. Annex 2b: Comparison between volumes measured on different MRIs and according to subgroup: In group 1 (G1: n=6), in both hippocampi, the D40% was < 7.4 Gy; in group 2 (G2: n=13), the Hcontra D40% was < 7.4 Gy; and in group 3 (G3: n=30), the D40% for both hippocampi was > 7.4 Gy. Annex 2c: Comparison between changes in volumes during the interval between MRIs and according to subgroup: In group 1 (G1: n=6), in both hippocampi, the D40% was < 7.4 Gy; in group 2 (G2: n=13), the Hcontra D40% was < 7.4 Gy; and in group 3 (G3: n=30), the D40% for both hippocampi was > 7.4 Gy. Annex 2d: Comparison between changes in % volumes during interval between MRIs and according to subgroup: In group 1 (G1: n=6), in both hippocampi, the D40% was < 7.4 Gy; in group 2 (G2: n=13), the Hcontra D40% was < 7.4 Gy; and in group 3 (G3: n=30), the D40% for both hippocampi was > 7.4 Gy [file 13014_2021_1835_MOESM2_ESM.docx]

Annex 2: Relevant comparison of hippocampal volumes their changes according to the time of the MRIs

Annex 2a: Comparison between volumes at a given MRI time according to subgroup: In group 1 (G1: n=6), in both hippocampi, the D_40%_ was < 7.4 Gy; in group 2 (G2: n=13), the H_contra_ D40% was < 7.4 Gy; and in group 3 (G3: n=30), the D_40%_ for both hippocampi was > 7.4 Gy

|  | At time of MRI_dosimetry_ | | | | | |
| --- | --- | --- | --- | --- | --- | --- |
|  | H_homo-G1_ | H_contra-G1_ | H_homo-G2_ | H_contra-G2_ | H_homo-G3_ | H_contra-G3_ |
| H_homo-G1_ |  | 0.41 | 0.28 |  | 0.03 |  |
| H_contra-G1_ | 0.41 |  |  | 0.97 |  | 0.65 |
| H_homo-G2_ | 0.28 |  |  | 0.75 | 0.14 |  |
| H_contra-G2_ |  | 0.97 | 0.75 |  |  | 0.61 |
| H_homo-G3_ | 0.03 |  | 0.14 |  |  | 0.01 |
| H_contra-G3_ |  | 0.65 |  | 0.61 | 0.01 |  |

|  | At time of MRI_relapse_ | | | | | |
| --- | --- | --- | --- | --- | --- | --- |
|  | H_homo-G1_ | H_contra-G1_ | H_homo-G2_ | H_contra-G2_ | H_homo-G3_ | H_contra-G3_ |
| H_homo-G1_ |  | 0.87 | 0.21 |  | 0.02 |  |
| H_contra-G1_ | 0.87 |  |  | 0.77 |  | 0.98 |
| H_homo-G2_ | 0.21 |  |  | 0.14 | 0.59 |  |
| H_contra-G2_ |  | 0.77 | 0.14 |  |  | 0.67 |
| H_homo-G3_ | 0.02 |  | 0.59 |  |  | 0.01 |
| H_contra-G3_ |  | 0.98 |  | 0.67 | 0.01 |  |

|  | At time of MRI_last_ | | | | | |
| --- | --- | --- | --- | --- | --- | --- |
|  | H_homo-G1_ | H_contra-G1_ | H_homo-G2_ | H_contra-G2_ | H_homo-G3_ | H_contra-G3_ |
| H_homo-G1_ |  | 0.41 | 0.11 |  | 0.01 |  |
| H_contra-G1_ | 0.41 |  |  | 0.73 |  | < 0.01 |
| H_homo-G2_ | 0.11 |  |  | 0.17 | 0.39 |  |
| H_contra-G2_ |  | 0.73 | 0.17 |  |  | 0.79 |
| H_homo-G3_ | 0.01 |  | 0.39 |  |  | 0.01 |
| H_contra-G3_ |  | < 0.01 |  | 0.79 | 0.01 |  |

Annex 2b: Comparison between volumes measured on different MRIs and according to subgroup: In group 1 (G1: n=6), in both hippocampi, the D_40%_ was < 7.4 Gy; in group 2 (G2: n=13), the H_contra_ D_40%_ was < 7.4 Gy; and in group 3 (G3: n=30), the D_40%_ for both hippocampi was > 7.4 Gy

|  | **Comparisons of volumes measured on MRI_dosimetric_ and MRI_relapse_** | | | | | |
| --- | --- | --- | --- | --- | --- | --- |
| *p* | H_homo-j0-G1_ | H_contra-j0-G1_ | H_homo-j0-G2_ | H_contra-j0-G2_ | H_homo-j0-G3_ | H_contra-j0-G3_ |
| H_homo-relapse-G1_ | 0.33 | 0.81 | 0.92 | 0.84 | 0.14 | 0.50 |
| H_contra-relapse-G1_ | 0.26 | 0.67 | 0.94 | 0.70 | 0.18 | 0.38 |
| H_homo-relapse-G2_ | **0.04** | 0.11 | 0.22 | 0.12 | 0.83 | **0.02** |
| H_contra-relapse-G2_ | 0.35 | 0.90 | 0.82 | 0.93 | 0.08 | 0.54 |
| H_homo-relapse-G3_ | **0.003** | **0.01** | **0.02** | **0.01** | 0.24 | **0.0004** |
| H_contra-relapse-G3_ | 0.18 | 0.56 | 0.90 | 0.59 | 0.07 | 0.11 |

|  | **Comparisons of volumes measured on MRI_relapse_ and MRI_last_** | | | | | | |
| --- | --- | --- | --- | --- | --- | --- | --- |
| *p* | H_homo-relapse-G1_ | H_contra-relapse-G1_ | H_homo-relapse-G2_ | H_contra-relapse-G2_ | H_homo-relapse-G3_ | H_contra-relapse-G3_ |  |
| H_homo-last-G1_ | 0.47 | 0.53 | 0.44 | 0.29 | 0.03 | 0.33 |  |
| H_contra-last-G1_ | 0.71 | 0.87 | 0.19 | 0.55 | **0.01** | 0.80 |  |
| H_homo-last-G2_ | 0.06 | 0.08 | 0.44 | **0.03** | 0.58 | **0.03** |  |
| H_contra-last-G2_ | 0.79 | 0.90 | 0.42 | 0.71 | 0.07 | 0.90 |  |
| H_homo-last-G3_ | **0.01** | **0.01** | 0.10 | **0.004** | 0.76 | **0.002** |  |
| H_contra-last-G3_ | 0.48 | 0.60 | 0.38 | 0.34 | **0.02** | 0.43 |  |

|  | **Comparisons of volumes measured on MRI_dosimetric_ and MRI_last_** | | | | | |
| --- | --- | --- | --- | --- | --- | --- |
| *p* | H_homo-j0-G1_ | H_contra-j0-G1_ | H_homo-j0-G2_ | H_contra-j0-G2_ | H_homo-j0-G3_ | H_contra-j0-G3_ |
| H_homo-last-G1_ | 0.08 | 0.23 | 0.46 | 0.24 | 0.27 | **0.02** |
| H_contra-last-G1_ | 0.15 | 0.45 | 0.79 | 0.48 | 0.09 | 0.053 |
| H_homo-last-G2_ | **0.01** | **0.03** | 0.06 | **0.03** | 0.55 | **0.005** |
| H_contra-last-G2_ | 0.28 | 0.63 | 0.85 | 0.66 | 0.32 | 0.86 |
| H_homo-last-G3_ | **0.002** | **0.003** | **0.01** | **0.003** | 0.14 | **0.0002** |
| H_contra-last-G3_ | 0.10 | 0.27 | 0.53 | 0.29 | 0.23 | **0.03** |

Annex 2c: Comparison between changes in volumes during the interval between MRIs and according to subgroup: In group 1 (G1: n=6), in both hippocampi, the D_40%_ was < 7.4 Gy; in group 2 (G2: n=13), the H_contra_ D_40%_ was < 7.4 Gy; and in group 3 (G3: n=30), the D_40%_ for both hippocampi was > 7.4 Gy

|  | **Comparison of changes between MRI_dosimetric_ and MRI_relapse_** | | | | | |
| --- | --- | --- | --- | --- | --- | --- |
| *p* | H_homo-G1_ | H_contra-G1_ | H_homo-G2_ | H_contra-G2_ | H_homo-G3_ | H_contra-G3_ |
| H_homo-G1_ |  | 0.43 | 0.92 | 0.24 | 0.74 | 0.66 |
| H_contra-G1_ | 0.43 |  | 0.25 | 0.56 | 0.12 | 0.50 |
| H_homo-G2_ | 0.92 | 0.25 |  | 0.09 | 0.78 | 0.45 |
| H_contra-G2_ | 0.24 | 0.56 | 0.09 |  | 0.03 | 0.14 |
| H_homo-G3_ | 0.74 | 0.12 | 0.78 | 0.03 |  | 0.22 |
| H_contra-G3_ | 0.66 | 0.50 | 0.45 | 0.14 | 0.22 |  |

|  | **Comparison of changes between MRI_relapse_ and MRI_last_** | | | | | |
| --- | --- | --- | --- | --- | --- | --- |
| *p* | H_homo-G1_ | H_contra-G1_ | H_homo-G2_ | H_contra-G2_ | H_homo-G3_ | H_contra-G3_ |
| H_homo-G1_ |  | 0.82 | 0.95 | 0.74 | 0.66 | 0.82 |
| H_contra-G1_ | 0.82 |  | 0.78 | 0.85 | 0.23 | 0.99 |
| H_homo-G2_ | 0.95 | 0.78 |  | 0.68 | 0.37 | 0.77 |
| H_contra-G2_ | 0.74 | 0.85 | 0.68 |  | 0.24 | 0.83 |
| H_homo-G3_ | 0.66 | 0.23 | 0.37 | 0.24 |  | 0.18 |
| H_contra-G3_ | 0.82 | 0.99 | 0.77 | 0.83 | 0.18 |  |

|  | **Comparison of changes between MRI_dosimetric_ and MRI_last_** | | | | | |
| --- | --- | --- | --- | --- | --- | --- |
| *p* | H_homo-G1_ | H_contra-G1_ | H_homo-G2_ | H_contra-G2_ | H_homo-G3_ | H_contra-G3_ |
| H_homo-G1_ |  | 0.42 | 0.91 | 0.39 | 0.84 | 0.70 |
| H_contra-G1_ | 0.42 |  | 0.14 | 0.89 | 0.32 | 0.39 |
| H_homo-G2_ | 0.91 | 0.14 |  | 0.13 | 0.04 | 0.37 |
| H_contra-G2_ | 0.39 | 0.89 | 0.13 |  | 0.07 | 0.36 |
| H_homo-G3_ | 0.84 | 0.07 | 0.87 | 0.07 |  | 0.18 |
| H_contra-G3_ | 0.70 | 0.39 | 0.37 | 0.36 | 0.18 |  |

Annex 2d: Comparison between changes in % volumes during interval between MRIs and according to subgroup: In group 1 (G1: n=6), in both hippocampi, the D_40%_ was < 7.4 Gy; in group 2 (G2: n=13), the H_contra_ D_40%_ was < 7.4 Gy; and in group 3 (G3: n=30), the D_40%_ for both hippocampi was > 7.4 Gy

|  | **Comparison of % of changes between MRI_dosimetric_ and MRI_relapse_** | | | | | |
| --- | --- | --- | --- | --- | --- | --- |
| *p* | H_homo-G1_ | H_contra-G1_ | H_homo-G2_ | H_contra-G2_ | H_homo-G3_ | H_contra-G3_ |
| H_homo-G1_ |  | 0.50 | 0.86 | 0.23 | 0.36 | 0.71 |
| H_contra-G1_ | 0.50 |  | 0.37 | 0.52 | 0.07 | 0.60 |
| H_homo-G2_ | 0.86 | 0.37 |  | 0.14 | 0.46 | 0.54 |
| H_contra-G2_ | 0.23 | 0.52 | 0.14 |  | 0.01 | 0.13 |
| H_homo-G3_ | 0.36 | 0.07 | 0.46 | 0.01 |  | 0.09 |
| H_contra-G3_ | 0.71 | 0.60 | 0.54 | 0.13 | 0.09 |  |

|  | **Comparison of % of changes between MRI_relapse_ and MRI_last_** | | | | | |
| --- | --- | --- | --- | --- | --- | --- |
| *p* | H_homo-G1_ | H_contra-G1_ | H_homo-G2_ | H_contra-G2_ | H_homo-G3_ | H_contra-G3_ |
| H_homo-G1_ |  | 0.89 | 0.87 | 0.90 | 0.33 | 0.87 |
| H_contra-G1_ | 0.89 |  | 0.67 | 1.00 | 0.10 | 0.98 |
| H_homo-G2_ | 0.87 | 0.67 |  | 0.72 | 0.28 | 0.65 |
| H_contra-G2_ | 0.90 | 1.00 | 0.72 |  | 0.16 | 0.98 |
| H_homo-G3_ | 0.33 | 0.10 | 0.28 | 0.16 |  | 0.09 |
| H_contra-G3_ | 0.87 | 0.98 | 0.65 | 0.98 | 0.09 |  |

|  | **Comparison of % of changes between MRI_dosimetric_ and MRI_last_** | | | | | |
| --- | --- | --- | --- | --- | --- | --- |
| *p* | H_homo-G1_ | H_contra-G1_ | H_homo-G2_ | H_contra-G2_ | H_homo-G3_ | H_contra-G3_ |
| H_homo-G1_ |  | 0.46 | 0.66 | 0.50 | 0.36 | 0.82 |
| H_contra-G1_ | 0.46 |  | 0.11 | 0.96 | 0.02 | 0.38 |
| H_homo-G2_ | 0.66 | 0.11 |  | 0.14 | 0.47 | 0.29 |
| H_contra-G2_ | 0.50 | 0.96 | 0.14 |  | 0.03 | 0.45 |
| H_homo-G3_ | 0.36 | 0.02 | 0.47 | 0.03 |  | 0.03 |
| H_contra-G3_ | 0.82 | 0.38 | 0.29 | 0.45 | 0.03 |  |
